# An automated skeleton extraction method for 3D point-cloud phenotyping of *Schima Superba* seedlings

**DOI:** 10.1371/journal.pone.0329715

**Published:** 2025-09-23

**Authors:** Qi Yikai, Zhou Yang, Xiang Longbin

**Affiliations:** 1 School of Intelligent Manufacturing and Energy Engineering, Zhejiang University of Science and Technology, Hangzhou, Zhejiang, China; 2 School of Artificial Intelligence and Information Engineering, Zhejiang University of Science and Technology, Hangzhou, Zhejiang, China; Guru Nanak College, INDIA

## Abstract

Aiming to address the issues of low efficiency and large errors in the manual measurement process of phenotypic parameters in *Schima Superba* seedlings, an automated non-destructive method for acquiring phenotypic parameters based on three-dimensional point clouds is proposed, which includes the main steps of alignment, skeleton extraction, and automatic phenotypic calculation. Aiming to overcome the technical challenges of stem and leaf separation in *Schima Superba*, a density-weighted voxel centroid method is proposed to extract skeleton points, combined with minimum spanning tree (MST) and principal component analysis (PCA) techniques to accurately identify the stem skeleton point cloud, effectively addressing the problem of stem-leaf separation. The separation process encounters difficulties at the stem-leaf junction, resulting in suboptimal separation accuracy. An improved K-means++ algorithm is proposed to initially estimate the number of adhering leaves based on coarse segmentation, followed by fine segmentation to achieve higher precision in leaf segmentation, effectively improving the accuracy and efficiency of the segmentation process. Following the completion of stem and leaf segmentation, a fully automated phenotypic characterization method based on the segmented point cloud is proposed for the first time. The method automatically outputs relevant phenotypic parameters, including plant height, stem length, stem diameter, and leaf area. The predicted correlation coefficients for the experimental phenotypes were 0.994, 0.992, 0.938, and 0.873, meeting the requirements for on-site measurement of phenotypic parameters in *Schima Superba* and providing strong technical support for plantation management and cultivar improvement.

## Introduction

*Schima Superba* is a prominent timber and ornamental tree species in China, valued for construction materials and silviculture due to its durable texture and decay resistance [1]. Accurate measurement of the phenotypic characteristics of *Schima Superba* seedlings is essential for forecasting their growth performance and ecological adaptations. Plant height is a critical indicator of growth rate and health, stem diameter reflects the plant’s mechanical strength and capacity for resource transport; larger diameters indicate more efficient water and nutrient transport, supporting greater leaf area and enhanced photosynthesis. Leaf area is considered a fundamental measure of a plant’s photosynthetic potential; larger leaf areas are associated with enhanced photosynthetic efficiency and carbon fixation capabilities, essential for maintaining ecological balance. Accurately measuring stem length is critical for understanding growth dynamics and branching patterns, which contributes to optimizing spatial layouts and planting densities. At present, the phenotypic measurement of *Schima Superba* seedlings still primarily relies on manual operation. However, manual operation is not only slow and inefficient but also incurs high labor costs and introduces significant errors. Therefore, the study of fully automated phenotypic parameter measurement of *Schima Superba* seedlings is an important research topic.

In recent years, numerous scholars and research teams have proposed 3D point cloud-based phenotypic measurements, with most studies focusing on economically and ecologically significant crops such as wheat, rice, and maize. However, a gap still exists in the relevant studies on *Schima Superba* seedlings. Therefore, the present study represents a novel attempt to address this gap, building on existing techniques. The analysis of phenotypic parameter extraction methods for these species provides valuable insights into studying *Schima Superba* seedlings. In plant phenotyping, the separation of stem and leaf components in point cloud data is crucial and is primarily achieved using machine learning and traditional skeletonization techniques. Machine learning methods rely heavily on specific stem and leaf features for classification. For instance, Paulus et al. [2] classified plant organs by color features, curvature-based regional growth segmentation, and Fast Point Feature Histograms (FPFH). Similarly, Liu et al. [3] utilized differences in vertical projected density between leaf and stem point clouds, while Paulus et al. [4] and Li et al. [5] computed local point features and used Markov Random Field (MRF) to differentiate stems and leaves. Dey et al. [6] used Conditional Random Field (CRF) to classify grape plants. These neural networks demonstrate high performance on laser-scanned images; however, they typically require manual operation with third-party software during preprocessing and cannot achieve full automation.

Alternative classification approaches include skeletonization techniques. Miao et al. [7] proposed a ring-corrected algorithm for plants with pendulous leaves. Wu et al. [8] developed a skeletonization technique using the Laplace transform to extract stem skeletons and measure phenotypic parameters such as leaf length and inclination. However, these methods are less effective for thin-stemmed *Schima Superba* seedlings. Zhou et al. [9] introduced a method combining vertical and horizontal slicing based on plant growth characteristics to facilitate stem-leaf separation by locating the main stem. Significant limitations persist in capturing the structural complexity of *Schima Superba* seedlings. Although methods like the raindrop algorithm by Zermas et al. [10] and the skeleton extraction and superpixel clustering by Dawei Li et al. [11] have shown high accuracy in some plants, their computational complexity and need for extensive preprocessing limit their applicability to *Schima Superba* seedlings. Xu et al. [12] utilized shortest path and clustering algorithms to extract tree skeletons effectively, applying breakpoint joining operations for broken branches up to two-thirds of the tree height. Hackenberg et al. [13] utilized clustering and cylindrical fitting techniques to extract cylindrical skeletons from tree point clouds. An L1 minimum spanning tree (L1-MST) algorithm was proposed by Mei et al. [14] to enhance tree skeleton extraction by combining the benefits of the L1 median skeleton and MST algorithms. However, the cylindrical fitting method is unsuitable for accurately capturing the skeletons of *Schima Superba* seedlings due to their thin and structurally intricate branches.

To address these challenges, a novel algorithm is proposed in this study to separate stems and leaves and extract phenotypic parameters for *Schima Superba* seedlings. The method begins with a density-weighted voxel centroid approach, combined with Minimum Spanning Tree (MST) construction and Principal Component Analysis (PCA) for precise stem skeleton point cloud extraction. Subsequently, Dijkstra’s shortest path algorithm, spatial search, and algorithms based on geometric feature analysis are employed to achieve stem and leaf separation. The leaf point cloud is then segmented using Locally Convex Connected Patches (LCCP) and an improved K-means++ algorithm, ensuring accurate parameter measurements. This innovative approach provides essential methods for effective plantation management and cultivar improvement, fostering advancements in related research.

## Materials and methods

### Image acquisition and experimental samples

**Fig 1:**
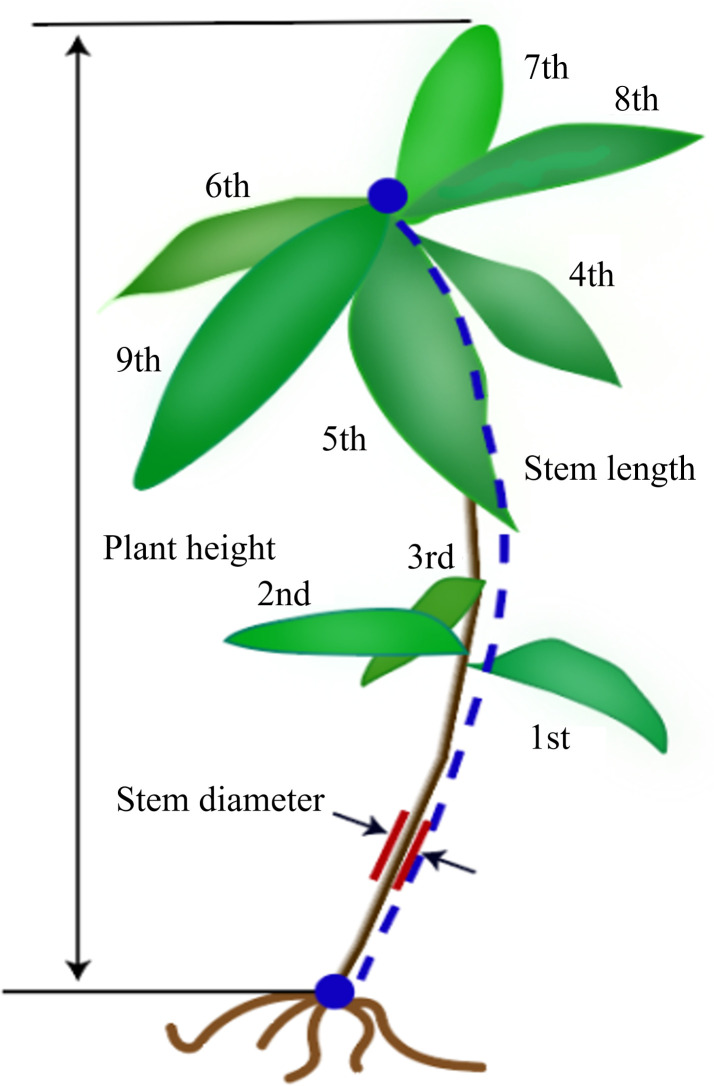
presents a graphical representation of the manual measurements of the height, stem length, and stem diameter of *Schima Superba* seedlings.

This study utilized a 3D image acquisition platform to automate the measurement of phenotypic parameters in *Schima Superba* seedlings. Detailed specifications of the platform are available in the literature published by our group [9]. The software in our study was upgraded to Microsoft Visual Studio 2022, PCL 1.13.1, and OpenCV 4.8.0, thereby providing more efficient data processing capabilities and enhanced image processing functions. A matte steel plate was also added to minimize light reflection and interference. During operation, point cloud images of the *Schima Superba* seedlings were acquired every 60 degrees. One hundred *Schima Superba* seedlings with heights ranging from 20 to 60 cm were selected, covering various growth stages. For manual measurements, plant height was defined as the distance from the potted *Schima Superba*’s soil surface to the seedling’s highest point, typically the tip of the tallest leaf. Stem diameter was measured at the mid-point of the stem, approximately 5 cm above the soil, using a vernier caliper. Stem length was measured from the soil surface to the tip of the stem, ensuring that the tape measure conformed to the curvature and natural form of the stem. (Fig 1) shows the manual measurement of height, stem length, and stem diameter of *Schima Superba* seedlings.

### Preprocessing

**Fig 2:**
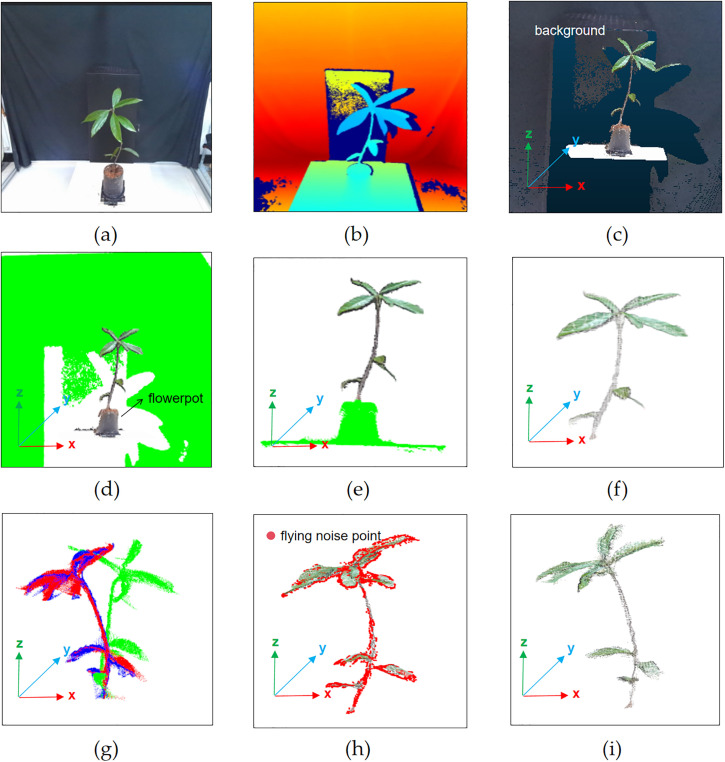
Preprocessing stages of the point cloud: (a) Real image; (b) Depth image; (c) Raw point cloud; (d) Background-removed point cloud; (e) Flowerpot-removed point cloud; (f) 0-degree aligned point cloud; (g) Fully aligned point cloud; (h) SOR-filtered point cloud, the red dots represent the removed time-of-flight noise; (i) Preprocessed aligned point cloud.

The raw point cloud captured by the camera includes background objects such as black iron plates, which can significantly interfere with leaf segmentation and plant phenotyping algorithms, leading to inaccurate results. Therefore, preprocessing is required for their removal and involves four steps: background removal, flowerpot removal, time-of-flight noise removal, and registration. The details of the algorithm are outlined in the literature published by our group [9], although the alignment process differs slightly. Fig 2(a) presents the actual image of the plant, while Fig 2(b) depicts the depth image. The raw point cloud of the plant is shown in Fig 2(c). After background removal, the background-removed point cloud is displayed in Fig 2(d); following the flowerpot removal, the flowerpot-removed point cloud is shown in Fig 2(e); and the 0-degree aligned point cloud is presented in Fig 2(f).

To generate a complete three-dimensional model of the *Schima Superba* seedlings, six multi-angle captured point clouds (0°, 60°, 120°, 180°, 240°, 300°) were utilized to complete the alignment. The pairwise aligned point clouds, numbered 1, 2, and 3, were created by aligning point clouds captured at adjacent angles (0°& 60°, 120°& 180°, 240°& 300°). Subsequently, point clouds 2 and 3 were aligned with point cloud 1, which served as the source point cloud, to form fully aligned point clouds 4 and 5. Finally, point clouds 4 and 5 were aligned to create the fully aligned point cloud of the *Schima Superba* seedlings. All alignment steps involved two phases: coarse alignment using the Four Point Congruent Sets (4PCS) algorithm [15] and fine alignment using the Iterative Closest Point (ICP) algorithm [16]. The resulting fully aligned point cloud is presented in Fig 2(g).

The 4PCS alignment algorithm was developed within the Random Sample Consensus (RANSAC) framework. The alignment process was accelerated by constructing and matching congruent four-point sets, thus reducing spatial matching operations. Following the initial alignment, a rotation-translation matrix was constructed using the ICP algorithm through iterative processes to identify corresponding point pairs between the source point cloud (P) and the target point cloud (Q). The source point cloud was transformed into the coordinate system of the target point cloud, and the distance error between the transformed source and target point clouds was then estimated. The above steps were repeated until the specified error threshold was met. Corresponding point pairs were determined by calculating the nearest neighbor for each point in the source point cloud relative to the target point cloud, thus achieving highly accurate alignment.

To verify the effectiveness of alignment, several experiments were conducted using criteria such as root mean square error (RMSE) of 0.00494659 m, average nearest-neighbor distance of 0.00377145 m, and computation time. Experimental results indicated that optimal alignment was achieved when the tolerance (Delta) was set to 0.01 and the expected overlap ratio was set to 0.7. It was evident that the point clouds captured from multiple directions were successfully aligned, resulting in a fully aligned point cloud.

Upon the completion of point cloud alignment, residual noise—potentially arising from sensor errors, environmental disturbances, or anomalies introduced during the data acquisition process—was still present. Statistical outlier removal (SOR) filtering [17] was applied to the fully aligned point cloud to eliminate this noise. The SOR filter effectively cleans the point cloud by removing points that deviate from the mean nearest-neighbor distance by more than a specified standard deviation. Experimentally, the SOR filter parameters were set to a K value of 100 and a standard deviation multiplier of 1.1, significantly reducing noise and decreasing the root-mean-square error (RMSE). The SOR-filtered point cloud of the *Schima Superba* is shown in Fig 2(h), while the final preprocessed and aligned point cloud is illustrated in Fig 2(i).

### Stem and leaf segmentation

Stem and leaf segmentation was a core step before extracting specific phenotypic parameters, consisting of four main steps. First, the overall skeleton points were extracted from the preprocessed aligned point cloud using the density-weighted centroid algorithm proposed in this study. Subsequently, a minimum spanning tree (MST) was constructed, and the highest junction nodes were filtered based on node types and Principal Component Analysis (PCA) results. These junction nodes were used to extract the stem skeleton points of the *Schima Superba* seedling. Then, using radius search, points belonging to the stem were identified within the aligned point cloud centered on the stem skeleton points, separating the stem point cloud from the leaf point cloud. Finally, the leaf point cloud was segmented using the local convexity connectivity determination (LCCP) method to identify adherent leaves. If adherent leaves were present, the K-Means++ algorithm was applied for fine segmentation, ultimately getting independent point clouds for each leaf.

### Skeletonization

By observing the *Schima Superba* seedlings’ point cloud data, it was found that the foliage of *Schima Superba* seedlings grows around the stem. The skeleton points were extracted using a density-weighted centroid algorithm. The octree approach [18] was employed for preliminary spatial subdivision, recursively dividing the space into smaller cubes. Simple geometric centroids often inaccurately position skeleton points due to noise in the point cloud data. For example, as shown in Fig 4(a), skeletons derived using the geometric center-of-mass method deviate from the actual morphology of the seedlings. To address these points, each small cube was filtered based on a density threshold. Only cubes exceeding this threshold are considered for subsequent weighted centroid calculations. A comparison between geometric and weighted centroids [19] is shown in Fig 4, where Fig 4(b) demonstrates the skeleton generated using the weighted centroid approach, which closely aligns with the proper morphology. The skeletonization process indirectly achieves down-sampling, enhancing resilience to noise and data density variations and improving accuracy while reducing data size.

**Fig 3:**
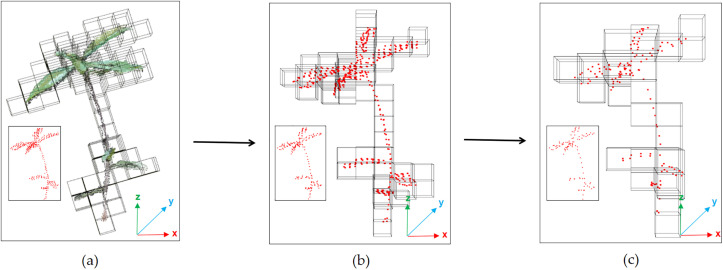
Skeletonization of the point cloud: (a) Weighted center of mass after the first skeletonization; (b) Weighted center of mass after the second skeletonization; (c) Weighted center of mass after the third skeletonization.

(1) The input point cloud data P={p_1_,p_2_,...,p_n_}, where n was the number of points, was decomposed into a set of cubes of size L × L × L.
(2) Each cube was divided spatially into eight equal sub-cubes V_j_.
(3) The number m_j_ of points within each sub-cube V_j_ was counted. If m_j_ exceeds the threshold θ, all points within the sub-cube are retained; otherwise, they are discarded.
(4) The geometric centroid of all points within the retained sub-cube was calculated. The points in all retained sub-cubes were then used as weights m_j_ to weight the geometric centroid C_j_ of the corresponding sub-cubes, forming a weighted centroid C_w_. The weighted centroid was regarded as a new point cloud.
(5) The above steps were repeated for the new point cloud, with each iteration doubling the edge length of the decomposed cubes from the previous cycle.

The initial cube size L was set to 0.01 m after several tests. Specifically, the geometric center C_j_ for each retained sub-cube V_j_ was calculated as follows:


$$C_{j}=\frac{1}{m_{j}}\sum_{i=1}^{m_{j}}p_{i}$$ (1)

Where p_i_ denotes the coordinates of the i point in the sub-cube V_j._ The weighted centroid C_w_ is calculated by taking the geometric centroid C_j_ of all retained sub-cubes and is given by:


$$C_{w}=\frac{\sum_{j=1}^{N}m_{j}\cdot C_{j}}{\sum_{j=1}^{N}m_{j}}$$ (2)

Where m_j_ represents the number of points within sub-cube V_j_, and C_j_ is the geometric centroid of sub-cube V_j_.

**Fig 4:**
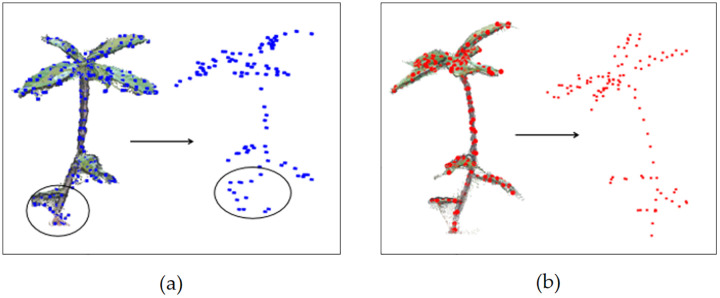
Comparison between the geometric centroid and skeletonization methods employed in this study. (a) depicts the skeleton extracted using the geometric centroid method, where the blue points represent the geometric center of mass skeleton points. (b) illustrates the skeletonization method employed in this study, with red points representing the skeleton points extracted by the proposed skeletonization method.

The weighted centroids corresponding to the first, second, and third skeletonizations are presented in Fig 3(a), Fig 3(b), and Fig 3(c), respectively. In this manner, the actual skeleton structure of *Schima Superba* seedlings was effectively represented, ensuring that the extracted skeleton points align closely with the actual morphology. The final comparison between the extracted skeleton points and the preprocessed point cloud is presented in Fig 4. Fig 4(b) illustrates the final comparison, effectively capturing details in the seedling point cloud data and improving the accuracy and robustness of skeleton extraction.

### Stem skeleton point extraction

Kruskal’s algorithm [20] was utilized to construct a minimum spanning tree(MST), enabling the accurate extraction of stem skeleton points from the overall skeleton points of *Schima Superba* seedlings. The nodes of the MST were then classified, followed by Principal Component Analysis (PCA) of the skeleton points to determine the principal structural direction of the overall skeleton points. The most significant bifurcation node along the principal direction was identified, serving as the endpoint for extracting the stem skeleton points.

**Fig 5:**
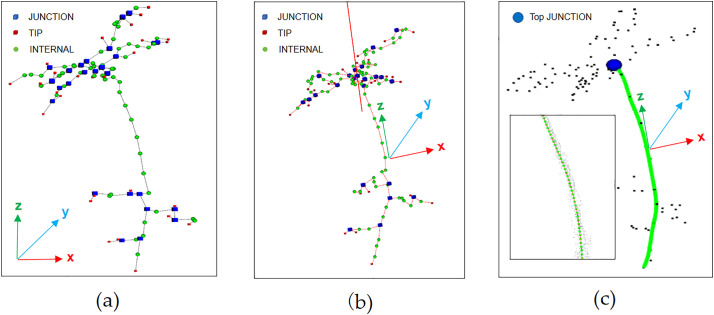
Illustration of the process for extracting stem skeleton points. (a) Minimum spanning tree with skeleton point connections and node classification; (b) PCA-derived principal direction line; (c) Visualization of the filtered highest forked node.

The specific process was as follows:

First, to connect the weighted centroid points extracted in Section 2.3.1 to reflect the skeleton structure of *Schima Superba* seedlings, the Kruskal algorithm was employed to construct the minimum spanning tree (MST)., as shown in Fig 5(a).

Next, the nodes in the MST were classified into three types [21]: TIP (leaf tip node): a node with no neighbors or only one neighbor, typically located at the tip of a *Schima Superba* seedling leaf. INTERNAL (internal node): a node with two neighbors, generally representing the middle part of the stem. JUNCTION (bifurcation node): a node with more than two neighbors, usually indicating a bifurcation point of the stem. Node classification results are shown in Fig 5(a).

After the classification of node types, PCA [22] was applied further to analyze the structural characteristics of *Schima Superba* seedlings. PCA was employed to transform potentially correlated variables into independent principal components. The first eigenvector of the point cloud data, representing the principal structural direction, was computed, normalized, and adjusted to align with the positive Y-axis. The principal direction line was determined and visualized with the point cloud’s center of mass, as shown in Fig 5(b). The main structural directions of the point cloud were thereby displayed, allowing for analysis of the stem’s structural characteristics within a standardized coordinate system.

As the stems of *Schima Superba* seedlings were typically erect, leaves and branches are generally concentrated near the stem, though they may deviate slightly. To ensure that the selected nodes were located along the stem, the JUNCTION nodes were identified within a 2 cm radius of the principal PCA direction line, as shown in Fig 5(b). Fig 5(c) highlights the JUNCTION node with a sphere.

### Stem point cloud segregation

To accurately extract the stem point cloud, Dijkstra’s shortest path algorithm [23] was applied to connect the highest bifurcation node to the root node across the skeleton point cloud, as shown in Fig 5(c). To address abrupt angle changes along the shortest path, cubic spline interpolation [24] generated a continuous and smooth skeletal stem curve. This smoothing process ensured that the extracted curve closely followed the primary structure of the stem.

**Fig 6:**
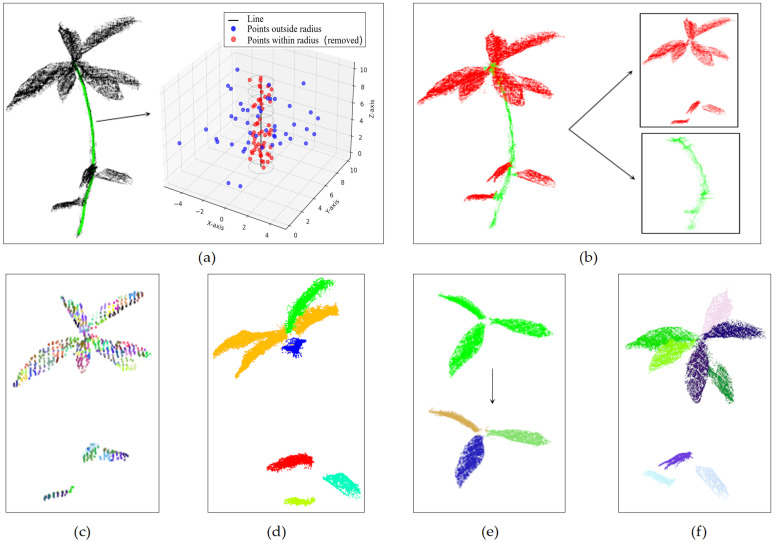
Process of stem and leaf segmentation. **(a)** Radius-based search for stem screening; **(b)** Separation of the stem point cloud; **(c)** Visualization of the separated stem; **(e)** Supersomal clustering process; **(f)** LCCP clustering process; **(g)** The upper image shows an incompletely segmented leaf map, while the lower image illustrates the results of K-means++ segmentation; **(h)** Final segmentation of the complete point cloud.

A KD-tree, a spatial search algorithm, was then employed to accelerate nearest-neighbor searching within a fixed radius. The search radius was set based on the manually measured stem diameter during preprocessing. For each point pair along the skeletal curve, neighboring points in the global point cloud were identified as stem points if their distances to the connecting line segment fell within the specified radius. This process effectively isolated the stem point cloud from other structures, such as leaves and stems. The stages of radius-based searching, stem segmentation, and leaf removal are depicted in Fig 6(a) and 6(b).

### Leaf segmentation

The overall leaf point cloud was obtained by subtracting the stem point cloud from the *Schima Superba* point cloud, with all leaves separated from the stem shown in Fig 6(b). Due to the complex geometry of leaf blades and their close connections, a single clustering method may not achieve the desired segmentation effect. Therefore, a Local Concavity-Conserving Clustering (LCCP) [25] method was applied for coarse segmentation of the overall leaf point cloud. The K-means++ algorithm [26] was subsequently applied for fine segmentation to improve segmentation accuracy and effectiveness for leaf point clouds where satisfactory segmentation was not achieved.

#### (1) Coarse segmentation.

Before LCCP segmentation, the overall leaf point cloud was initially over-segmented using the Voxel Cloud Connectivity Segmentation (VCCS) method [27], as shown in Fig 6(c). VCCS generated hyper voxels using a local region-growing variant of K-means clustering and stored the resulting neighborhood graph in an octree data structure. The octree resolution (R_voxel_) and seed grid resolution (R_seed_) were adjusted to ensure uniform growth and connectivity of hypervoxels. This adjustment efficiently pre-segmented the leaf point cloud, preparing it for subsequent LCCP segmentation.

This study considered only the point cloud of colorless *Schima Superba* seedling leaves. A 37-dimensional feature space, including curvature values, XYZ coordinates, and a 33-dimensional Fast Point Feature Histogram (FPFH) [28], was used for clustering, with similarity distances between neighboring voxels calculated as follows:


$$D=\sqrt{\frac{w_{c}D_{c}^{2}}{c_{\max^{2}}}+\frac{w_{s}D_{s}^{2}}{3R_{seed}^{2}}+w_{n}D_{n}^{2}}$$ (3)

where D represents the similarity distance between neighboring voxels; D_c_ represents the curvature difference between voxels; D_n_ represents the FPFH spatial distance using the histogram intersection kernel; D_s_ represents the spatial Euclidean distance between voxels; R_seed_ is the seed resolution; C_max_ is the maximal curvature in a voxel’s neighborhood; and w_c_, w_s_, and w_n_ are the weights for curvature, spatial distance, and geometric similarity, respectively. The parameters were set as follows: R_voxel_ = 0.008m, R_seed_ = 0.01m, w_c_ = 0.2, w_s_ = 0.4, w_n_ = 1. This similarity metric guided the flow clustering process, where voxels were iteratively assigned to hypervoxels. During clustering, each point was assigned a unique marker representing its cluster. The distance between a voxel and the center of its hyper voxel was calculated using Equation (3), and neighboring voxels were iteratively included until no further neighbors satisfied the similarity threshold.

Next, LCCP clusters the over-segmented point cloud based on concavity-convexity relationships, as shown in Fig 6(d). CC and SC determine the concavity-convexity relationships between clusters. The parameters for implementing the LCCP clustering algorithm in PCL were set as follows: connecting crystal centers; defining the LCCP splitter, setting the concavity threshold to β_th_ = 20 and the smoothness threshold to θ_th_ = 0.1, and setting the minimum segmentation size to 0. Some clusters contained only a small number of points or outliers; thus, only target clusters with more than 100 points were retained.

#### (2) Fragmentation.

Although LCCP segmentation effectively handled most leaves, it might still fail to fully separate neighboring leaves due to the complexity of leaf morphology. After LCCP segmentation, any remaining unsegmented leaves were further refined using the K-means++ algorithm [29]. This study proposes an optimization for manually determining the number of clusters K in K-means++ clustering, as described in the literature [26]. The value of K is adaptively determined using the following method. The unsegmented leaves are shown in the upper part of Fig 6(e), while the K-means++ segmentation results are displayed in the lower part of Fig 6(e). First, the average number of points $\bar{\mu }$ per leaf was calculated:


$$\bar{\mu }=\frac{1}{n}\sum_{i=1}^{n}S_{i}$$ (4)

Si is the number of points in the i leaf and n is the total number of leaves.

Subsequently, leaves with a point count exceeding the mean value $\bar{\mu }$ were selected for further processing. Fine clustering was conducted on the leaves using the K-means++ algorithm, an unsupervised machine-learning method. In the traditional K-means algorithm, the results often vary depending on the selection of initial clustering centers, resulting in instability and slow convergence. As a result, an optimized K-means++ algorithm was employed to efficiently and effectively select appropriate initial clustering centers.

The number of clusters K was set as the integer part of the point count of an anomalous leaf divided by the average point count, calculated as follows:


$$K=\left\lfloor \frac{S_{e}}{\bar{\mu }}\right\rfloor$$ (5)

Where S_e_ represents the point count of each anomalous leaf, if the calculated K value was less than 2, further clustering was not performed to preserve its original structure. The maximum number of iterations was set to 100, the convergence threshold to 0.1 cm, and the minimum distance between initial cluster centers was 0.2 cm. Each part of the leaf was fully separated while preserving the leaf structure, as shown in Fig 6(f).

### Morphological feature extraction

#### 1. Plant height.

Plant height was defined as the distance from the base to the top of a *Schima Superba* seedling as follows:


$$H=Y_{\max}-Y_{min}$$ (6)

**Fig 7:**
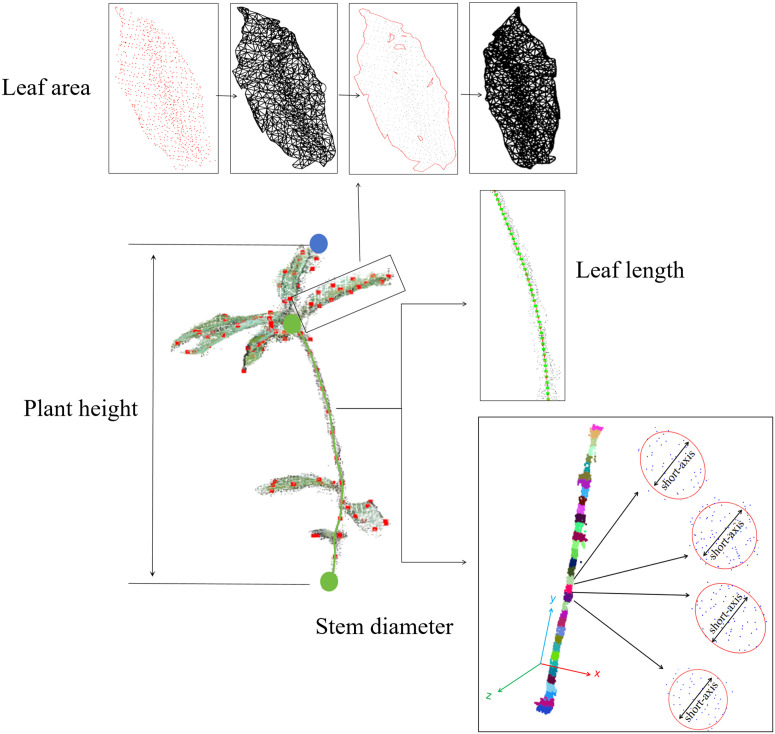
Illustration of the process for extracting stem skeleton points. **(a)** Schematic diagram representing plant height; **(b)** Plot showing stem diameter measurements obtained by slicing and fitting ellipses; **(c)** Visualization of stem length measurements; **(d)** Visualization of the blade area hole repair process. From left to right: the blade point cloud; the triangular cross-section converted into a surface triangular mesh; the repaired hole; and the triangular mesh of the repaired blade.

Y_max_ and Y_min_ denote the Y coordinates of the highest and lowest points along the Y-axis of the point cloud obtained from preprocessing. Fig 7 illustrates the schematic diagram of the plant height calculation.

#### 2. Stem length.

Stem length was the total path distance calculated from the plant root’ to the highest bifurcation node. The procedure for calculating the stem length was outlined: First, the filtered highest bifurcation node was linked to the root node through Dijkstra’s algorithm, as illustrated in Fig 7(b). The resulting shortest path was subsequently smoothed by applying cubic spline interpolation. The Euclidean distances between consecutive points along the interpolated curve are calculated, and the stem length is obtained by summing these distances. This approach ensures a precise and continuous representation of the stem length.

#### 3. Stem diameter.

Stem diameter was defined as the average cross-sectional width of the stem in *Schima Superba* seedlings, representing its thickness. The calculation procedure was outlined as follows: Initially, PCA was applied to the stem point cloud to determine the principal axis of the stem, ensuring a global alignment of the data. Next, 1-cm interval slices were extracted along the principal axis, starting 5 cm above the stem base to minimize interference from the soil. The points in each selected slice were projected onto the XOZ plane, and ellipses were fitted to these projected points [30] to extract the lengths of their minor semi-axes. To obtain a stable and accurate stem diameter, four slices within 5 cm above the soil were selected, and the average length of their minor semi-axes was calculated as the final stem diameter, as shown in Fig 7. These slices were explicitly chosen to minimize interference from soil and foliage, thereby ensuring accurate measurements of stem thickness. This method was considered reliable and robust for determining the stem diameter.

#### 4. Leaf area.

As shown in Fig 7(d), the independent leaf point clouds were converted into triangular surface meshes using the triangle dissection method. Internal holes were introduced in this process when neighboring points exceeded a predefined search radius. To minimize hole formation while preserving edge details, a balanced strategy was employed to control the search radius and repair the holes, improving leaf area measurement accuracy. The hole repair process begins with boundary identification, where edges traversed only once are detected by analyzing the boundary points of the triangular mesh. The maximum ring extraction algorithm then distinguishes internal holes from external boundaries by labelling the largest ring as the maximum and all other rings as internal holes. After the boundaries of the internal holes were identified, a point-link hole repair algorithm was applied to patch the holes, iteratively generating new triangles until the holes were filled. The areas of all triangles in the patched mesh were summed and divided by two to yield the estimated area of each leaf. This method provides an accurate estimate of the leaf area while preserving the two-sided nature of the triangular mesh. To verify the accuracy of the area calculation, each leaf was scanned by Sanner, with A4 paper size used as a reference, after binarized, and the area of the black pixels was calculated as the actual leaf area.

## Results

**Fig 8:**
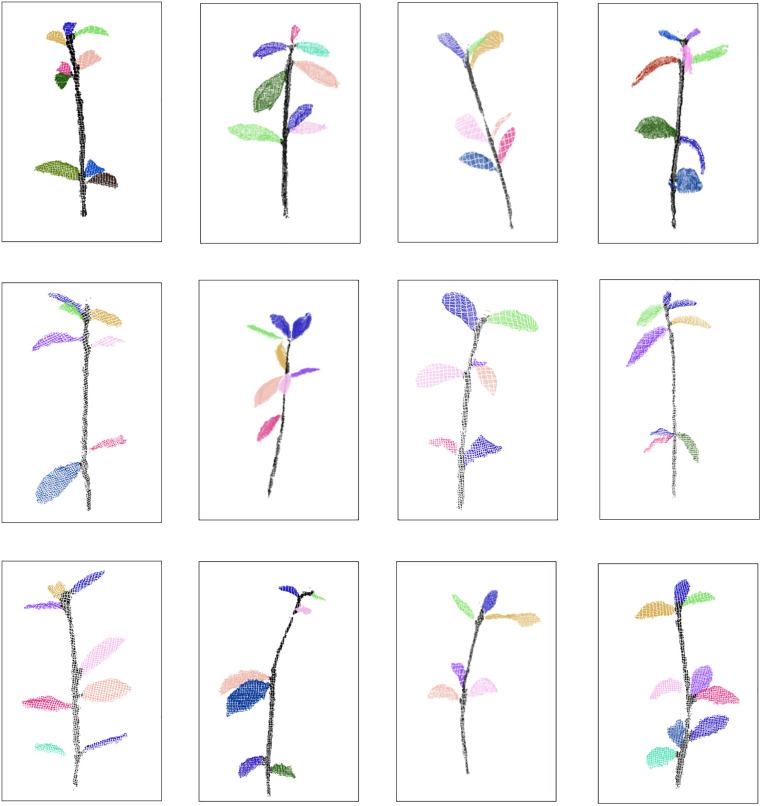
An example of a typical sample split involving 12 plants, where different colors represent distinct leaves after segmentation.

In this study, 12 randomly selected samples of *Schima Superba* seedlings were used to evaluate the segmentation performance of the proposed method, as shown in Fig 8. Different colours represent the segmented single-leaf point cloud data, and the 3D surface model generated after segmentation is visually presented. The segmentation results closely align with manual measurements.

### Accuracy assessment

**Fig 9:**
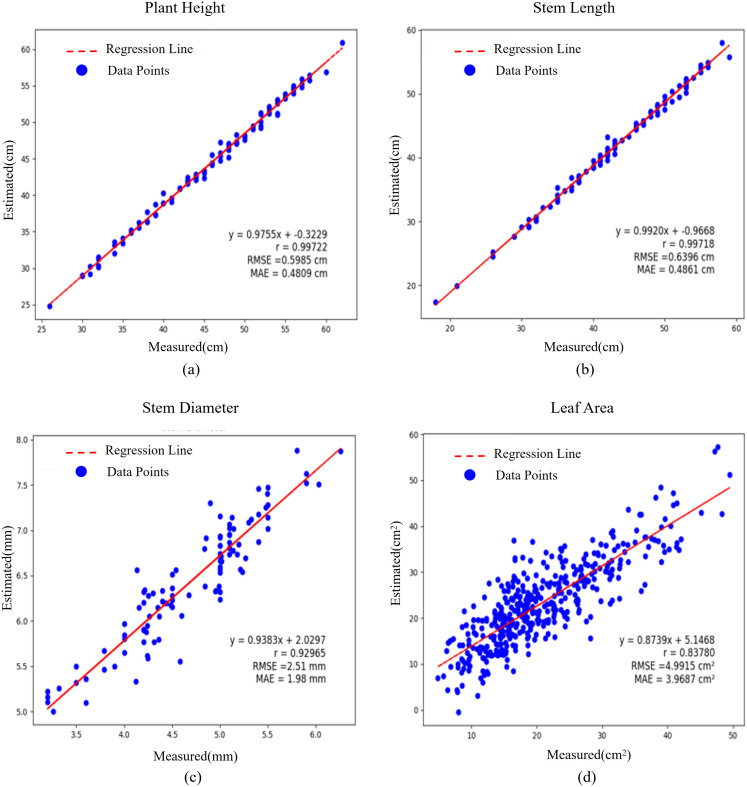
Comparison of four phenotypic parameters: manual measurements and systematic estimates. The (a-d) regression line represents a straight line fitted to the estimates. An r = 1 indicates that the estimates perfectly match the actual values and serve as the reference line.

As shown in Fig 9, linear regression analysis was performed on 100 *Schima Superba* seedlings to assess the correlation between manual measurements and system estimates. The correlation coefficient (r) and root mean square error (RMSE) were calculated to quantify system performance. Measurement statistics are presented in Table 1.

**Table 1:** Statistics of measured indicators.

Measurement Indicator	Correlation coefficient	RMSE	MAE	Average of manually measured values	Average of systematic estimates
Plant height(cm)	0.994	0.599	0.4809	42.64	44.12
Stem length(cm)	0.992	0.639	0.4861	41.07	39.82
Stem diameter(mm)	0.938	2.561	1.98	4.56	4.19
Leaf area(cm²)	0.874	4.991	3.969	23.41	20.65

The results indicated that plant height and stem length were accurately measured, achieving correlation coefficients of 0.994 and 0.992 and root mean square errors (RMSEs) of 0.599 cm and 0.639 cm, respectively. These findings demonstrate that the system’s estimations were highly consistent with manual measurements. A correlation coefficient of 0.938 and an RMSE of 2.561 mm were achieved for stem diameter. The primary sources of error were incomplete removal of flight noise during denoising and inaccuracies in the point cloud alignment process. These issues caused stem alignment errors during ellipsoid fitting, as leaf point clouds occupied a higher weight, affecting parameter extraction accuracy. Nevertheless, the correlation coefficients suggest that the results are adequate for practical applications.

A correlation coefficient of 0.874 and an RMSE of 4.991 cm² were observed for leaf area. This error was partly attributed to the incomplete separation of stem-leaf junctions, which caused portions of the stem point cloud to be misclassified as leaves. Furthermore, bias may have been introduced by the validation method for manually measuring leaf area. The process involved copying the leaf tillers onto A4 paper using a printer and calculating the manual value of leaf area by multiplying the ratio of non-zero pixels to the total number of pixels by the area of the paper. During the copying process, leaf flattening likely caused the manual measurements to be slightly larger than the actual values, leading to lower system estimates.

Overall, the phenotypic measurements showed only slight deviations from actual values, and the correlation coefficients and other indicators were sufficient for practical applications.

To validate the method’s feasibility, a sensitivity analysis was conducted on two core parameters of the density-weighted voxel centroid skeletonization algorithm: voxel size L and density threshold θ. The parameter L dictates the spatial discretization granularity. Smaller values of L capture finer structural details but amplify noise sensitivity and computational load; conversely, larger values result in the loss of structural details. The parameter θ filters data based on sub-voxel point counts. Higher values of θ reduce noise interference but may truncate skeletons in low-density regions, whereas lower values introduce noise artifacts.

As detailed in Table 2, L was tested at 0.0025 m increments within the range of 0.005 m to 0.015 m, while θ was evaluated at 3-point intervals from 3 to 15 points. Each parameter combination was applied to a 20-sample dataset. Performance was quantified by average stem segmentation accuracy using a controlled-variable approach.

**Table 2:** Sensitivity analysis of L and θ parameters.

Parameter Name	Value	Average Stem Segmentation Accuracy	Optimal Value
**Voxel Size L (m)**	0.005	78.4%	**0.010**
	0.0075	89.7%	
	**0.010**	**96.1%**	
	0.0125	88.2%	
	0.015	75.6%	
**Density Threshold θ (pts)**	3	82.5%	**6**
	**6**	**95.8%**	
	9	90.3%	
	12	84.7%	
	15	76.9%	

The sensitivity analysis revealed distinct performance patterns across parameter values. At L = 0.005 m, irregular skeleton oscillations were observed due to noise interference, yielding 78.4% accuracy. Conversely, L = 0.015 m resulted in excessive smoothing with structural detail loss (75.6% accuracy). Optimal balance was achieved at L = 0.010 m, maintaining 96.1% accuracy through effective detail preservation and noise suppression. Regarding density threshold, θ = 3 produced spurious skeleton branches (“burrs”) with 82.5% accuracy, while θ = 15 caused premature skeleton termination at stem tips (76.9% accuracy). The θ = 6 configuration optimally filtered noise while retaining critical features (95.8% accuracy).

Consequently, the parameter combination L = 0.010 m and θ = 6 points was identified as optimal. This configuration enables accurate stem morphology reconstruction with effective noise suppression and detail retention, establishing a robust foundation for subsequent stem-leaf segmentation and validating the method’s robustness.

For leaf segmentation refinement using K-means++ clustering, an adaptive strategy was implemented to determine the number of clusters K, eliminating the requirement for fixed preset values or sensitivity analysis. Specifically, the average number of points per leaf obtained from LCCP segmentation was calculated to screen aberrant leaves. The K value was dynamically computed for each qualified leaf. Remaining parameters—including maximum iterations (100), convergence threshold (0.1 cm), and minimum initial center spacing (0.2 cm)—were set according to general stability criteria, with robustness experimentally confirmed. This adaptive mechanism avoids biases introduced by subjective K presetting, thereby enhancing segmentation accuracy in complex foliar structures.

A 10-fold cross-validation (k = 10) was performed using 100 *Schima Superba* seedling samples. The dataset was randomly partitioned into 10 subsets. During each iteration, nine subsets (90 samples) were utilized for algorithm parameter optimization, while the remaining subset (10 samples) served as an independent test set. This procedure was repeated 10 times, ensuring every sample was tested once. The design balances computational efficiency with statistical validity, leveraging available data to assess methodological stability.

**Fig 10:**
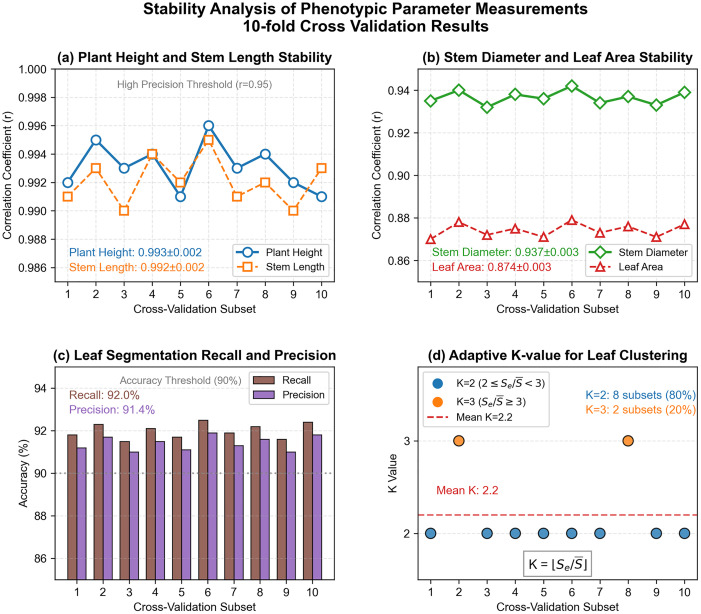
Stability analysis of phenotypic parameter measurements 10-fold cross-validation results.

Stability analysis results are presented in Fig 10: (a) Plant height and stem length correlation stability are shown with a blue solid line (circles) representing plant height (r), orange dotted line (squares) for stem length (r), and gray dotted line indicating the high-precision threshold (r = 0.95), where plant height = 0.993 ± 0.002 and stem length = 0.992 ± 0.002 (mean ± SD); (b) Stem diameter and leaf area stability are depicted with green solid line (diamonds) for stem diameter (r) and red dashed line (triangles) for leaf area (r), showing stem diameter = 0.937 ± 0.003 and leaf area = 0.874 ± 0.003 (mean ± SD); (c) Comparative recall and precision metrics for leaf segmentation are represented by brown bars (recall: correctly segmented leaves/total leaves) and purple bars (precision: correctly segmented leaves/algorithm-output leaves), with gray dashed line marking the 90% precision threshold, yielding average recall = 92.0% and precision = 91.4%; (d) Adaptive K-value distribution for leaf clustering displays blue dots (K = 2, 80%) and orange dots (K = 3, 20%), with red dashed line indicating the mean value (2.2).

### Cross-validation result analysis

#### 1. Stability of phenotypic parameter measurements.

For plant height and stem length, correlation coefficients exceeding 0.990 were demonstrated across all 10 subsets, with mean values of 0.993 and 0.992 (SD = 0.002) respectively and variations below 0.5%. RMSE and MAE measurements were consistent with baseline validation outcomes (Table 1), exhibiting standard deviations under 0.015 cm, thereby confirming algorithmic insensitivity to subset partitioning and high stability. Regarding stem diameter and leaf area, mean correlation coefficients of 0.937 (SD = 0.003) and 0.874 (SD = 0.003) were observed with approximately 3% variation. While stem diameter RMSE (2.5 mm) was influenced by point cloud weighting during alignment, leaf area RMSE (4.98 cm²) corresponded to baseline validation results (4.99 cm²), collectively indicating minimal influence of data partitioning on methodological reliability.

#### 2. Robustness of stem-leaf separation precision.

Recall (92.0% ± 0.3%) and precision (91.4% ± 0.3%) consistently exceeded the 90% threshold across all ten subsets, demonstrating that the stem-leaf segmentation algorithm maintains high consistency between samples and thereby confirming the efficacy of the skeleton extraction and clustering strategies.

#### 3. Reasonableness of the adaptive K-value determination method.

In 20–60 cm *Schima Superba* seedlings, the main stem is distinct during the vegetative growth stage, with leaves surrounding it. Each plant contains 4–15 leaves, stem point clouds constitute 35–45% of the data, and leaf point clouds exhibit an ellipsoidal distribution. The adaptive K-value mechanism dynamically aligns with the structural characteristics of plants: At scale ratios of 2–3 (80% of samples), K = 2 is assigned when leaf sizes are relatively uniform; at scale ratios ≥3 (20% of samples, primarily 55–60 cm specimens), K = 3 accommodates significant heterogeneity in leaf size (i.e., the coexistence of substantially larger and smaller leaves). This approach eliminates the over-segmentation observed in 20–30 cm seedlings and the under-segmentation observed in 40–60 cm seedlings using fixed-K methods.

Statistical analyses reveal a mean K-value of 2.2 (SD = 0.4), a significant linear correlation with seedling height, stem thickness, and leaf area (Pearson’s r = 0.89, p < 0.01), and a combined error rate 56–67% lower than that of the fixed K-value method across the 20–60 cm range. It captures the developmental morphology of plants without manual adjustment, making it suitable for large-scale seedling phenotyping.

#### 4. Consistency with basic validation.

The mean values from cross-validation showed a high degree of consistency with the basic validation results presented in Table 1 (e.g., plant height: r = 0.994; leaf area: r = 0.874), indicating that 10-fold division introduced no systematic bias and that the original conclusions are representative. The fluctuation ranges (e.g., plant height: r = 0.991–0.996) reflect the minor impacts of individual samples, which fall within the error tolerance of the method.

## Discussion

In this study, an automated 3D point cloud phenotypic parameter measurement method for *Schima Superba* seedlings was developed by non-destructively processing image data collected by a 3D image acquisition device. Due to *Schima Superba*’s complex phenotypic characteristics, relatively few studies have been conducted on this plant, especially in the area of stem-leaf separation. In this study, we focused on addressing this critical step of stem-leaf separation.

**Fig 11:**
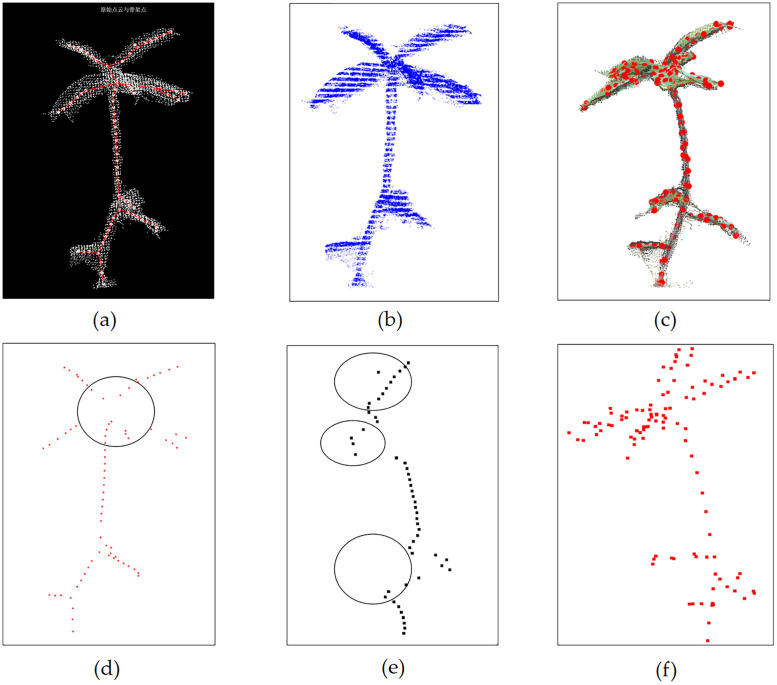
Comparison of three different skeletonization methods. **(a)** Skeletonization using the Laplace method; **(b)** Skeletonization based on sliced clustering; **(c)** Skeletonization method proposed in this study; **(d)** Skeleton points obtained using the Laplace method; **(e)** Skeleton points obtained through sliced clustering; **(f)** Skeleton points obtained through the skeletonization method proposed in this study.

Skeletonization is a key step in stem-leaf separation, and three skeletonization methods were compared in this study. First, the Laplace skeletonization method proposed by Teng Miao et al. (2021) (Fig 11(a) and 11(d)) was ineffective in dealing with the complex structure at the bifurcation point of the *Schima Superba* seedling, failing to extract the skeleton of the stem and leaves accurately. Second, the slice clustering method proposed by Xiang et al. (2019) (Fig 11(b) and 11(e)) simplified multiple leaves into a single skeleton point in dense leaf regions, resulting in the loss of detailed leaf structure. This simplification process also led to a significant decrease in the skeletonization accuracy of larger or scattered leaves.

To address these issues, this study proposes a density-weighted voxel centroid method, which performs excellently in handling stem bifurcation points and complex leaf structures, significantly improving skeleton extraction accuracy. In addition, while manual setting of the K-value for the K-means++ algorithm was used in Reference [26], this study improves upon this by incorporating an automated K-value determination method, which enhances leaf segmentation accuracy and resolves the difficulty of manually setting the number of clusters in traditional methods.

In addition to the methodological advantages outlined above, the performance of the method with respect to computational efficiency, robustness to environmental noise, and cross-species scalability is also noteworthy.

Computational efficiency: The method proposed in this study employs a density-weighted voxel centroid skeletonization algorithm combined with minimum spanning tree (MST) topology analysis, enabling stem-leaf segmentation for an individual *Schima Superba* seedling to be completed in mainstream CPU environments without reliance on GPU-based graphic acceleration. Minimal computational resources are consumed by the algorithm, rendering it suitable for deployment in low-power computing scenarios. In comparison with deep learning models, its advantages primarily stem from a reduced dependency on data labeling, thereby avoiding the need for large-scale training samples and high-performance computing resources.

With respect to robustness to environmental noise, the method proposed in this study enhances adaptability to partial occlusion via a multi-view point cloud fusion strategy. When the stem of a *Schima Superba* seedling is partially occluded by leaves, a more complete 3D reconstruction of the stem can still be achieved by increasing the shooting angle and fusing multi-view data. The density-weighted voxel centroid skeletonization algorithm is inherently adaptive to variations in point cloud density, whereas the LCCP (Locally Convex Connected Patches) + improved K-means++ leaf segmentation method remains effective for mild leaf folding. Thus, in general, the algorithm proposed in this study exhibits good robustness. However, the accuracy of leaf phenotypic parameter extraction may be affected in scenarios involving leaf folding caused by extreme external forces—a limitation that current alternative algorithms also fail to address.

With respect to cross-species scalability, the present method is applicable to plants possessing a single main stem with leaves growing around it. Moreover, it can be transferred to tree species (e.g., oak, camphor) that are morphologically similar to *Schima Superba* seedlings. However, parameter optimization and algorithmic fine-tuning are still required for species exhibiting significant differences in leaf topology—an area that represents a key direction for subsequent research.

While the methods described above exhibit significant advantages, their limitations require further investigation. Limitations in the extraction of phenotypic parameters for *Schima Superba* seedlings primarily stem from the inability to completely eliminate certain errors. Since time-of-flight noise cannot be fully eliminated, and a portion of effective edge points must be discarded during point cloud denoising, the algorithm proposed in this study introduces certain baseline errors in parameter estimation. Achieving complete and accurate segmentation of stem-leaf junctions remains challenging during stem-leaf separation, with residual stem point clouds often remaining in the leaf point cloud—an issue that directly impacts the accuracy of leaf parameter estimation. Errors in stem length measurement arise from two factors: deviation of the stem skeleton fitting curve from the actual morphology, and offset of the surface datum extracted via direct-pass filtering due to uneven soil planes. Errors in stem diameter primarily arise from the point cloud alignment process, in which the numerical dominance of leaf point clouds skews the weights of the alignment matrix toward leaves, reducing stem alignment accuracy and impairing ellipse fitting results. Nevertheless, systematic experimental results demonstrate that the final outcomes still meet the requirements of practical applications.

The measurement accuracy of the proposed method is compared with that of other methods in the literature. Reference [30] utilized 3D point cloud data to automatically extract plant height, stem diameter, leaf angle, and leaf area of sorghum. The RMSE for plant height obtained by their method is 2.0715 higher than that measured by our proposed method. Reference [31] developed a method for capturing 3D phenotypic data of leafy vegetables using smartphone video recordings to measure plant height, leaf number, leaf length, and leaf inclination. Compared to the RMSE for plant height in their study, our method shows an improvement of 1.2215. In Reference [32], a clustering-based algorithm (such as K-means or DBSCAN) was used to separate the stem and leaves of maize and measure plant morphological features. Our method shows improvements in RMSE for stem length (0.1434), stem diameter (0.69), and plant height (1.1015). Reference [33] employed time-of-flight 3D imaging technology to measure field maize, including plant height, leaf inclination, and stem diameter. Our method improves the RMSE by 2.778 for stem diameter and 5.2015 for plant height. Reference [34] proposed a model-based 3D point cloud segmentation method for sunflower phenotyping. The point cloud was generated using structured light technology to segment the stem, petiole, and leaves, and leaf area was calculated using NURBS reconstruction. Our method improves the MAE for leaf area by 0.0085. Reference [35] proposed a framework based on multi-view stereo point clouds for automatic segmentation and extraction of leaf phenotypic features from Maranta arundinacea and Dieffenbachia picta, aiming to calculate leaf area, length, width, and inclination. The MAE for leaf area in their study is similar to that of our method. Overall, our method demonstrates lower errors and advantages in measuring plant height, stem length, and leaf area. In contrast, the measurement error for stem diameter is consistent with that of other methods.

However, certain systematic errors are unavoidable in manual measurement methods. Ensuring an absolute vertical reference for the tape measure during plant height measurement is challenging; physical pressure from the vernier caliper during stem diameter measurement may cause deformation of young stems; and during leaf area measurement, detached leaves undergo passive expansion when flattened by the scanner, resulting in scanned areas that exceed their in situ actual areas. Overall, manual measurements tend to overestimate values, which results in the relative underestimation of point cloud-reconstructed leaf area data in absolute terms. While correlation coefficients and other indicators validate the reliability and applicability of the scheme, the potential impact of manual measurement processes on validation results should be acknowledged.

Additionally, although the LCCP and K-means++ algorithms have enhanced the performance of leaf segmentation, the adaptive clustering strategy still requires refinement when handling leaves with extreme morphologies. Future research will focus on optimizing algorithmic environmental adaptability, cross-species scalability, and the integration of deep learning techniques to further enhance the universality and accuracy of the methods.

## Conclusions

In this study, a low-cost 3D phenotyping system was constructed using an Azure Kinect camera, and an automated measurement method was proposed for four phenotypic parameters of *Schima Superba* seedlings in the height range of 20–60 cm. The results indicated that the plant height and stem length estimated by the system were highly correlated with manually measured values (r > 0.992). In contrast, the correlation coefficients for stem diameter and leaf area were slightly lower but remained at a high level (r > 0.873). The experimental results provide an efficient and economical solution for plant phenotypic characterization, improving measurement accuracy and efficiency through automated techniques while reducing common human errors associated with traditional methods. Future work will focus on optimizing the applicability and stability of the algorithm, particularly under varying environmental conditions. Plans include the development of smarter algorithms, the expansion to a broader range of plant species, and the exploration of deep learning techniques to enhance segmentation and recognition accuracy.

## Abbreviations

3D: Three-dimensional
PCL: Point Cloud Library
RGB-D: Red, Green, Blue, and Depth
TIP: Leaf tip node
INTERNAL: Internal node
JUNCTION: Bifurcation node
ICP: lterative Closet Point
LCCP: Locally Convex Connected Patches
MST: Minimum Spanning Tree
PCA: Principal Component Analysis
4PCS: Four Point Congruent Sets
SOR: Statistical outlier removal filtering
VCCS: Voxel Cloud Connectivity Segmentation
CRF: Conditional Random Field
MRF: Markov Random Field
FPFH: Fast Point Feature Histogram
L1-MST: L1 minimum spanning tree
TP: True Positive
FP: False Positive
FN: False Negative
Acc: Stem-Leaf Separation Accuracy
MAE: Mean Absolute Error
RMSE: Root Mean Squared Error
DBSCAN: Density-Based Spatial Clustering of Applications with Noise
RANSAC: Random Sample Consensus
KD-tree: K-dimensional tree.
